# Poly(ADP-ribose)polymerase-1 modulates microglial responses to amyloid β

**DOI:** 10.1186/1742-2094-8-152

**Published:** 2011-11-03

**Authors:** Tiina M Kauppinen, Sang Won Suh, Youichirou Higashi, Ari E Berman, Carole Escartin, Seok Joon Won, Chao Wang, Seo-Hyun Cho, Li Gan, Raymond A Swanson

**Affiliations:** 1 Department of Neurology, University of California, San Francisco, and Veterans Affairs Medical Center, 4150 Clement Street (127), San Francisco, CA 94121, USA; 2 Gladstone Institute of Neurological Disease, Department of Neurology, University of California, 1650 Owens Street, San Francisco, CA 94158, USA

**Keywords:** Alzheimer's disease, beta amyloid peptide, calbindin, cytokines, microglia, NF-κB, poly(ADP-ribose)polymerase-1, trophic factors

## Abstract

**Background:**

Amyloid β (Aβ) accumulates in Alzheimer's disease (AD) brain. Microglial activation also occurs in AD, and this inflammatory response may contribute to disease progression. Microglial activation can be induced by Aβ, but the mechanisms by which this occurs have not been defined. The nuclear enzyme poly(ADP-ribose) polymerase-1 (PARP-1) regulates microglial activation in response to several stimuli through its interactions with the transcription factor, NF-κB. The purpose of this study was to evaluate whether PARP-1 activation is involved in Aβ-induced microglial activation, and whether PARP-1 inhibition can modify microglial responses to Aβ.

**Methods:**

hAPP_J20 _mice, which accumulate Aβ with ageing, were crossed with PARP-1^-/- ^mice to assess the effects of PARP-1 depletion on microglial activation, hippocampal synaptic integrity, and cognitive function. Aβ peptide was also injected into brain of wt and PARP-1^-/- ^mice to directly determine the effects of PARP-1 on Aβ-induced microglial activation. The effect of PARP-1 on Aβ-induced microglial cytokine production and neurotoxicity was evaluated in primary microglia cultures and in microglia-neuron co-cultures, utilizing PARP-1^-/- ^cells and a PARP-1 inhibitor. NF-κB activation was evaluated in microglia infected with a lentivirus reporter gene.

**Results:**

The hAPP_J20 _mice developed microglial activation, reduced hippocampal CA1 calbindin expression, and impaired novel object recognition by age 6 months. All of these features were attenuated in hAPP_J20_/*PARP-1^-/- ^*mice. Similarly, Aβ_1-42 _injected into mouse brain produced a robust microglial response in wild-type mice, and this was blocked in mice lacking PARP-1 expression or activity. Studies using microglial cultures showed that PARP-1 activity was required for Aβ-induced NF-κB activation, morphological transformation, NO release, TNFα release, and neurotoxicity. Conversely, PARP-1 inhibition increased release of the neurotrophic factors TGFβ and VEGF, and did not impair microglial phagocytosis of Aβ peptide.

**Conclusions:**

These results identify PARP-1 as a requisite and previously unrecognized factor in Aβ-induced microglial activation, and suggest that the effects of PARP-1 are mediated, at least in part, by its interactions with NF-κB. The suppression of Aβ-induced microglial activation and neurotoxicity by PARP-1 inhibition suggests this approach could be useful in AD and other disorders in which microglial neurotoxicity may contribute.

## Background

The accumulation of beta amyloid (Aβ) peptide contributes to disease pathogenesis in Alzheimer's disease (AD) [1,2]. Aβ induces microglial activation under experimental conditions, and microglial activation may in turn lead to neuronal loss and cognitive decline in AD [3]. However, microglial activation is not a univalent state, but instead encompasses a variety of morphological, biochemical, and secretory responses [4], many of which can occur independently of one another [5-7]. Activated microglia can release NO, proteases, and other neurotoxic factors, but they can also release certain neurotrophic factors and clear Aβ plaques and fibrils by phagocytosis [8-11]. Epidemiological studies suggest that anti-inflammatory drugs may reduce AD incidence [12], but in a randomized controlled trial, non steroidal anti-inflammatory therapy did not slow cognitive decline in AD [13]. Thus, the net effect of microglial activation in AD remains unresolved, and it is possible that interventions selectively targeting neurotoxic aspects of microglial activation may be more effective than broad-spectrum anti-inflammatory approaches.

Poly(ADP-ribose) polymerase-1 (PARP-1) is a nuclear protein that regulates cellular inflammatory responses through interactions with several transcription factors [14,15]. In particular, PARP-1 interaction with NF-κB has been identified as a major factor regulating macrophage and microglial activation [14,16-18]. Auto poly(ADP-ribosyl)ation of PARP-1 enhances the formation of the NF-κB transcription complex by dissociating NF-κB p50 from PARP-1 and thereby allowing NF-κB to bind to its DNA binding sites [19-21]. PARP-1 can also bind to the p65 NF-κB subunit [22,23]. Both PARP-1 gene deficiency and PARP-1 inhibitors prevent the morphological changes associated with microglial activation, and suppress microglial release of proteases, NO, and cytokines [16,17,19,24,25]. PARP-1 activation occurs in human AD [26], but the role of PARP-1 activation in microglial responses to Aβ is not known.

In this study we characterize the effects of PARP-1 inhibition and gene deletion on Aβ-induced microglial activation, and show that these effects are mediated, at least in part, through PARP-1 regulation of NF-κB. PARP-1 inhibition in microglial cultures reduced Aβ-induced release of NO and TNFα and prevented neurotoxicity, but did not impair microglial uptake of Aβ peptides. In vivo studies confirmed that PARP-1 gene depletion reduces Aβ-induced microglial activation, and studies in mice expressing human amyloid precursor protein with familial AD mutations (hAPP_J20 _mice) showed ameliorated neuronal and behavioral deficits when crossed to *PARP-1^-/- ^*mice. These results suggest that PARP-1 inhibition reduces deleterious effects of Aβ-induced microglial activation.

## Methods

### Materials

Cell culture reagents were obtained from Cellgro/Mediatech (Herndon, VA), unless otherwise stated. Culture plates (24-well plates) and 75 cm^2 ^polystyrene culture flasks were from Falcon/Becton Dickinson (Franklin Lakes, NJ). N-(6-oxo-5,6-dihydrophenanthridin-2-yl)-N,N-dimethylacetamide (PJ34) was obtained from Sigma. (E)-3-(4-methylphenylsulfonyl)-2-propenenenitrile (BAY 11-7082) was obtained from Alexis Biochemicals. Amyloid beta _1-42 _(Aβ), reverse amyloid beta _42-1 _(rAβ), and carboxyfluorescein (FAM)-labeled amyloid beta _1-42 _(FAM-Aβ), were obtained from Biopeptide Co. Inc. (San Diego, CA). Primary antibodies used were: rabbit polyclonal anti-poly(ADP-ribose) (PAR; Trevigen, Gaithersburg, MD), rabbit polyclonal anti-mouse ionized calcium binding adapter molecule 1(Iba-1; Waco), rabbit polyclonal anti-glial fibrillic acid protein (GFAP; Chemicon, Temecula, CA), rabbit polyclonal anti-microtubule-associated protein 2 (MAP2; Chemicon, Temecula, CA), mouse monoclonal anti-amyloid β 3D6 (Elan Pharmaceuticals) and rabbit polyclonal anti-Calbindin D-28k (Swant, Bellinzona, Switzerland). Secondary antibodies used were: anti-rabbit IgG conjugated with Alexa Fluor 488 or 594 (Molecular Probes Inc., Eugene, OR).

### Mice

All animal studies were approved by the San Francisco Veterans Affairs Medical Center animal studies committee and follow NIH guidelines. *PARP-1^-/- ^*mice were derived from the 29S-Adprt1^tm1Zqw ^strain, originally developed by Z. Q. Wang [27], and obtained from Jackson Laboratory (Bar Harbor, ME). *PARP-1^-/- ^*mice used for cell culture studies were backcrossed for over 10 generations with wt CD-1 mice, and wt CD-1 mice were used as their controls. *PARP-1^-/- ^*mice used for *in vivo *studies and for generating the hAPP_J20_/*PARP-1^-/- ^*mice were backcrossed to the C57BL/6 strain for over 10 generations. The hAPP_J20 _mice on the C57BL/6 background were obtained from Dr. Lennart Mucke (Gladstone Institute). These mice express a hAPP minigene with the familial AD-linked Swedish (K670N, M671L) and Indiana (V717F) mutations, under control of the platelet-derived growth factor (PDGF) β-chain promoter [28]. The hAPP_J20 _mice were crossed with the *PARP-1^-/- ^*mice to obtain the breeder genotypes: *PARP^+/- ^*and hAPP_J20 _/*PARP-1^+/-^*. These were in turn crossed to generate subsequent generation breeder genotype mice along with the four genotypes of interest: wt, *PARP-1^-/-^*, hAPP_J20 _and hAPP_J20_/*PARP-1^-/-^*. Male mice 5 - 6 months of age were used for in vivo studies. Genotype was re-confirmed on each mouse using tissue obtained at euthanasia.

### Neuron cultures

Neuron cultures were prepared as described previously [29]. In brief, cortices were removed from embryonic day 16 wt mice, dissociated into Eagle's minimal essential medium (MEM) containing 10 mM glucose and supplemented with 10% fetal bovine serum (Hyclone, Ogden UT) and 2 mM glutamine, and plated on poly-D-lysine-coated 24-well plates at a density of 7 × 10^5 ^cells per well. After 2 days *in vitro*, 22 μM cytosine β-D-arabinofuranoside (Sigma, St. Louis, MO) was added to inhibit the growth of non-neuron cells. After 24 hours, the medium was removed and replaced with a 1:1 mixture of glial conditioned medium (GCM) and MEM. This medium was 50% exchanged with fresh medium after 5 days. The cultures contained about 97% neurons and 3% astrocytes as assessed by immunostaining for the neuron marker MAP2 and the astrocyte marker GFAP.

### Microglia and microglia-neuron co-cultures

Cortices were dissected from 1-day old mice and dissociated by mincing followed by incubation in papain (40 units) and DNase (2 mg) for 10 minutes at 37°C. After centrifugation for 5 minutes at 500 g, the cells were re-suspended and triturated with a fire-polished Pasteur pipette into Eagle's minimal essential medium (MEM) containing 5 mM glucose and supplemented with 10% fetal bovine serum (Hyclone, Ogden UT) and 2 mM glutamine. Cells were plated on 24-well plates or glass coverslips at a density of 2 × 10^5 ^cells per well, or in 75 cm^2 ^flasks at a density of 5 × 10^6 ^cells per flask, and maintained in a 37°C in a 5% CO_2 _incubator. The medium was changed at 3 days *in vitro *and once per week thereafter. These cultures contained both astrocytes and microglia. Microglia were isolated from these cultures at age 2 to 3 weeks in vitro by shaking, and collecting the floating cells [24]. The cells were re-plated at a density of 5 × 10^5 ^cells per well in 24-well plates for microglial monocultures, or at the density of 5 × 10^4 ^cells per well on top of 6-day *in vitro *neuron cultures in 24-well plates for microglia-neuron co-cultures. The purity of the re-plated microglial monocultures was > 99%, and the microglia-neuron co-cultures contained about 7% microglia, 90% neurons, and 3% astrocytes as assessed by immunostaining for the microglial marker Iba-1, the neuron marker MAP2 and the astrocyte marker GFAP.

### Preparation of Aβ

For in vitro use, 1 mM stock solutions of Aβ peptides (Aβ and rAβ) were diluted to 250 μM with MEM and incubated for 1 hour at 37°C to produce a mixture of Aβ monomers and oligomers [30]. For in vivo use Aβ peptides were diluted to 1 mg/ml (220 μM) with normal saline. The solution was prepared within one hour of use and kept at room temperature in order to maintain the peptides in oligomeric form (fibrils would block the syringe) [30,31].

### Cell culture treatments

Neuron monocultures and microglia-neuron co-cultures were used at neuron day 7 *in vitro*. Microglial cultures were used at day 2-3 after re-plating. Cultures were incubated with 5 μM of Aβ or 5 μM of rAβ alone, or with inhibitors of PARP activation (PJ34, 400 nM) or NF-κB activation (BAY 11-7082, 5 μM) for the designated intervals. In some experiments, 5 μM of carboxyfluorescein-labeled amyloid β_1-42 _(FAM-Aβ) was used to detect microglial phagocytosis of Aβ fibrils. All compounds were dissolved in MEM (microglia) or GCM/MEM mixture (neurons), and these solutions were used alone for control conditions.

### Microglia activation, neurotoxicity, and phagocytosis in vitro

All evaluations in this study were performed by observers blinded to the experimental conditions. Neuronal survival was determined by cell counting in 5 randomly selected phase contrast microscopic fields per culture well. Values were normalized to counts in control wells from the same 24-well plate. Microglia morphology was assessed by phase contrast microscopy of unfixed cells. Microglia with two or more thin processes were considered as ramified, resting microglia, and microglia with less than two processes, or with amoeboid cell soma, were classified as activated [24]. The numbers of resting and activated microglia were counted in 5 randomly selected fields per culture well. Immunostaining was performed with cultures fixed with 1:1 methanol:acetone at 4°C. Cultures were characterized with antibodies to GFAP and Iba-1 as previously described [24]. Antibody binding was visualized with suitable Alexa Fluor - conjugated anti-IgG. Negative controls were prepared by omitting the primary antibodies. For detection of poly(ADP-ribose), cultures were incubated with rabbit antibody to PAR. Microglial phagocytosis of Aβ was imaged using three-dimensional confocal imaging of cultures with microglia-astrocyte co-cultures exposed to 5 μM of FAM-Aβ. Microglial phagocytic activity in microglial monocultures was quantified as described [32] with minor modifications by measuring FAM fluorescence remaining in the cells after two washes with MEM. Nonspecific Aβ adherence to the culture plate surface was evaluated by measuring FAM fluorescence in cell-free culture wells that had been incubated with FAM-Aβ for 24 hours.

### Nitric oxide, cytokine and trophic factor measurements

Microglial cultures were placed in 250 μl of MEM and incubated with Aβ or rAβ for 24 hours. Nitric oxide production was measured by using Griess reagent as previously described [25]. Cytokines and tropic factors were analyzed in 50 μl aliquots of cell culture medium using a Milliplex mouse multiplex immunoassay bead system according to the manufacturer's instructions (Millipore). Each sample was assayed in duplicate, and the fluorescent signal corresponding to each cytokine was measured with a BioPlex 200 system (Bio-Rad, Hercules, CA) in parallel with known standards. Nonspecific interactions between beads and test compounds were screened by running the immunoassay with test compounds dissolved in medium without cell culture exposure. The reverse sequence Aβ_42-1 _(but not Aβ_1-42_) was found to interfere with the assay in a non-specific manner, and thus rAβ-treated cultures could not be analyzed. Values for cytokine and trophic factor assays were normalized to the protein content of each well as determined by the bicinchoninic assay [33].

### Microglial NF-κB activity

Microglia were infected with lentivirus encoding destabilized, enhanced green fluorescence protein driven by the NF-κB promoter (Lenti-κB-dEGFP) [34] at 8-9 days *in v*itro, while still in co-culture with astrocytes. Infection was performed in culture medium with viral titer of 6.4 × 10^-8 ^pg of p24 antigen/ml. The microglia were isolated and re-plated 5-6 days later, and used for experiments 2 days after re-plating. Photographs were prepared at the designated intervals after Aβ exposure, and the percent of cells expressing green fluorescent protein (GFP) were counted in five random fields within each well.

### Intracerebral amyloid-β injections

Wt and PARP-1^-/- ^mice were given stereotaxic injections of Aβ, rAβ, or saline vehicle into hippocampus (anteroposterior 2.0 mm, mediolateral 1.5 mm, and dorsoventral 2.0 mm from bregma and cortical surface) with a Hamilton syringe. Mice received 1 μg of Aβ (or rAβ) in a 1 μl injection volume. Injections were made over a 5 minute period and the needle was withdrawn after an additional 5 minutes. Some animals received i.p. injection of PARP inhibitor (PJ34, 15 mg/kg) 15 minutes prior the Aβ injections. In a subset of experiments FAM-Aβ was used to confirm uniform injection volumes and identify the area Aβ diffusion. Mice were euthanatized 6 hours after Aβ injections, and brains were removed after transcardial perfusion with a 0.9% saline and 4% formaldehyde. Brains were post-fixed in 4% formaldehyde overnight, cryoprotected by immersion in 20% sucrose for 24 hours, and stored at -80°C.

### Brain immunostaining and cytokine measurements

One hemisphere (forebrain) was removed after saline perfusion, frozen, and stored at -80°C for biochemical studies. The remaining hemisphere was post-fixed in 4% formaldehyde, cryoprotected in sucrose, and cryostat sectioned into 30 μm coronal sections for immunostaining. Immunostaining was performed with 30 μm coronal sections as described previously [25,35]. Microglia were stained using Iba-1 antibody, Aβ plaques were stained with 3D6 antibody and calbindin expression was detected with Calbindin D-28k antibody. Primary antibody staining was visualized with suitable goat anti-IgG antibody conjugated with either Alexa Fluor 594 or 488. Brain sections were mounted on cover slips with DAPI-labeled mounting media (Vectashield) to facilitate recognition of brain structures. Negative controls were prepared by omitting the primary antibodies. Microscope imaging settings were kept uniform for all samples. Microglial morphology was analyzed in hippocampal CA1 and DG areas and in perirhinal cortex, with the exception of Aβ-injected brains, where microglial morphology was evaluated in 250 × 200 μm area starting 100 μm lateral to the needle track. Microglial activation was scored according to morphology and cell number (Table 1), as modified from [25]. Calbindin expression was determined by measuring the mean optical density in the designated, uniform-sized regions of interest with the ImageJ program (NIH). Values were measured on three comparable sections from each mouse, background values were subtracted, the resulting values averaged to give one value per mouse. For cytokine assays (Milliplex multiplex assays, Millipore) the forebrain hemispheres were homogenized 1:3 weigh to volume in M/PIER Mammalian Protein reagent (Thermo Scientific) with complete protease inhibitor (Sigma), following by centrifugation. Cytokine levels determined using standards in each assay plate, and values were normalized to protein content of the supernatants.

**Table 1:** Scoring for microglial activation

Cell shape (% with activated morphology)	Score
0%	**0**

1-25%	**1**

26-69%	**2**

≥70%	**3**

**Cell number** (cells per 50 mm^2^)	**Score**

1-5	**1**

6-11	**2**

12-17	**3**

18-28	**4**

29-39	**5**

≥ 40	**6**

Microglia were identified by Iba-1 immunoreactivity. Scoring with the two criteria were combined to yield an aggregate microglia activation score (0-9).

### Quantification of Aβ

The lysates used for cytokine assay were further processed with guanidine buffer. ELISAs were performed as described [36] and normalized to total protein content. We used antibodies that recognize species referred to as Aβ_1-42 _and Aβ_1-X _(Elan Pharmaceuticals). The Aβ_1-42 _ELISA detects only Aβ_1-42_, and the Aβ_1-X _ELISA detects Aβ_1-40_, Aβ_1-42_, and Aβ_1-43_, as well as C-terminally truncated forms of Aβ containing amino acids 1-28.

### Behavioral testing

Novel object recognition was tested in a white square plastic chamber 35 cm in diameter under a red light, as previously described [37]. Mice were transferred to the test room and acclimated for at least 1 hour. On the first day, mice were first habituated to the testing arena for 15 minutes and then each mouse was presented with two identical objects in the same chamber and allowed to explore freely for 10 min a training. On the second day, mice were placed back into the same arena for the 10 min test session, during which they were presented with an exact replica of one of the objects used during training (familiar object) and with a novel, unfamiliar object of different shape and texture. Object locations were kept constant during training and test sessions for any given mouse. Arenas and objects were cleaned with 70% ethanol between each mouse. Frequency of object interactions and time spent exploring each object was recorded with an EthoVision video tracking system (Noldus Information Technology, Leesbug, VA). Frequency of object interactions was used for analyses.

Spatial learning and memory were tested by the Morris Water Maze test, using a circular pool (122 cm in diameter, filled with opaque water at 24°C as describe previously [25,35]. The mice were trained first to locate a platform with a visible cue (days 1 - 2), and then to locate a hidden platform (days 3 - 5) using large spatial cues in the room. The platform was moved to a new quadrant in each session during the visible platform cue training. The platform remained in the same quadrant throughout all the sessions during hidden platform training. The mice received two training sessions per day for five consecutive days. Each session consisted of three one-minute trials with a 10-minute inter-trial interval. The interval between the two daily sessions was 3 hours. Once the mice located the platform they were allowed to remain on it for 10 seconds. Mice that failed to find the platform within one minute were manually placed on the platform for 15 seconds. Time to reach the platform (latency), distance traveled (path length), and swim speed (velocity) were recorded with a video tracking system (Noldus).

### Statistical analysis

For in vivo studies, the "n" denotes the number of mice in each group, and for cell culture studies the "n" denotes the number of independent experiments, each performed in triplicate or quadruplicate. All data are expressed as the mean ± SEM. Microglial morphological changes were evaluated with the Kruskal-Wallis test followed by the Dunn's test for multiple group comparisons. Data form Morris Water Maze test was analyzed by repeated measures one-way ANOVA. All other data were compared with ANOVA followed by the Bonferroni's test for multiple group comparisons.

## Results

### Effects of PARP-1 deficiency in hAPP_J20 _mice

**Figure 1:**
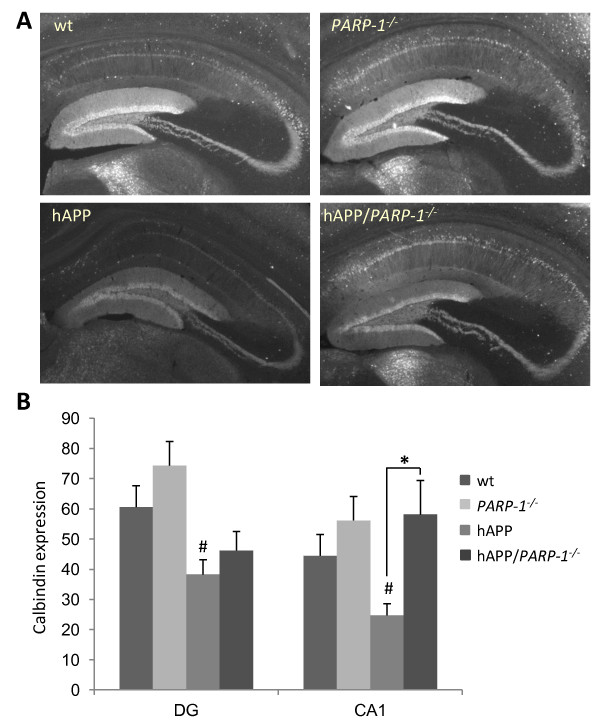
**PARP-1 deficiency preserves calbindin expression in hAPP_J20 _mice**. A, Photomicrographs from hippocampus of 6 month-old mice shows calbindin staining in the molecular layer of DG and pyramidal cell layer of CA1. Quantified data (mean density) are shown in panel (B). * p < 0.05; # p < 0.05, versus wt. n = 9-11.

**Figure 2:**
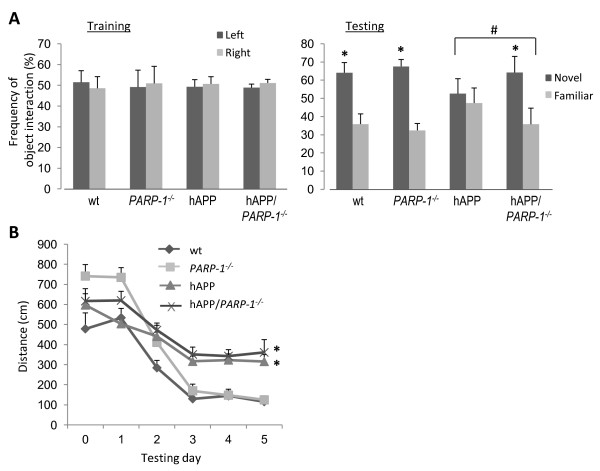
**Effect of PARP-1 gene deficiency on cognitive performance in hAPP_J20 _mice**. A, Object recognition memory, as measured by the percentage of visits to a familiar versus a novel object. Both the left and right objects are novel during the training session. n = 8-12; * p < 0.05 vs. familiar object, ^#^p <.05 between the indicated groups. B, Spatial learning and memory as assessed by the Morris water maze test on sequential testing days. * p < 0.05 vs. wt; n = 8-12.

The hAPP_J20 _mouse expresses human amyloid precursor protein with AD-linked mutations [28]. The hAPP_J20 _mice were crossed with *PARP-1^-/- ^*mice to evaluate the effects of PARP-1 gene deletion in this mouse model of AD. Spatial memory decline in hAPP_J20 _mice correlates with loss of calbindin in the hippocampus [38]. A loss of calbindin in the hAPP mouse hippocampus was likewise observed in the present study (Figure 1A). This loss was attenuated in the hippocampal CA1 pyramidal layer of the hAPP_J20_/*PARP-1^-/- ^*mice, but not in the dentate gyrus (Figure 1). Cognitive testing confirmed deficits in the hAPP_J20 _mice as assessed by both the novel object recognition test and the Morris water maze test of spatial memory (Figure 2). The hAPP_J20_/*PARP-1^-/- ^*mice performed better than the hAPP_J20 _mice in the novel object recognition test, but not in the Morris water maze test (Figure 2).

**Figure 3:**
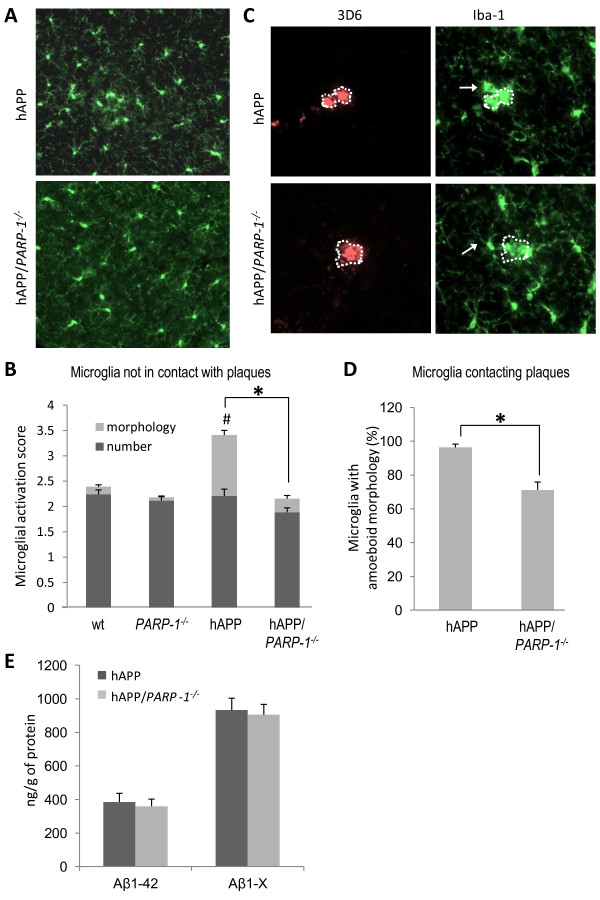
**PARP-1 deficiency reduces microglial activation but not Aβ accumulation in hAPP_J20 _mice**. Microglia in cortex of 6-month old hAPP_J20 _and hAPP_J20 _/*PARP-1^-/- ^*mouse are immunostained with Iba-1 (green). The microglia had less activated morphology in the hAPP _J20 _/*PARP-1^-/- ^*brains, in both non-plaque areas (A) and in microglia contacting Aβ plaques (3D staining, red) (C). Dotted line shows the margins of plaque area. Arrows show microglia with differing morphologies; amoeboid, lacking visible processes in hAPP_J20 _brain, and non-amoeboid with visible processes in the hAPP_J20_/*PARP-1^-/- ^*brain. B, Quantification of microglia activation without plaque contact is presented in stacked columns presenting the scores for both microglial number and morphology. D, Morphological quantification of microglia contacting plaques. * p < 0.05; # p < 0.05, versus wt. n = 9-11. E, ELISA measurements of Aβ_1-42 _and Aβ_1- X_. n = 8-12.

The hAPP_J20 _mice exhibit Aβ accumulation and scattered amyloid plaque formation by age 6 months [28]. These mice also show accumulation of amoeboid microglia at the amyloid plaques, and increased number of activated microglia throughout cortex and hippocampus (Figure 3). Despite comparable levels of Aβ accumulation in hAPP_J20 _and hAPP_J20_/*PARP-1^-/- ^*mice (Figure 3E), microglial activation was reduced in the hAPP_J20_/*PARP-1^-/- ^*mice, in both amyloid plaques and in non-plaque areas (Figure 3). The total number of microglia was not statistically different between genotypes, in either amyloid plaque areas (hAPP_J20 _vs. hAPP_J20_/*PARP-1^-/-^*; 7.06 ± 0.94 vs. 6.22 ± 1.36 cells per mm^2^) or in non-plaque areas (Figure 3).

Cytokine levels in the hAPP_J20 _mouse brains were not significantly different than in wt brains, but some cytokines were altered in the *PARP-1^-/- ^*and the hAPP_J20_/*PARP-1^-/- ^*brains (Table 2).

**Table 2:** Cytokine levels in mouse brain

	wt	*PARP-1^-/-^*	hAPP	hAPP/*PARP-1^-/-^*
IP-10	38.2 ± 2.5	62.5 ± 7.7 *	48.9 ± 6.7	87.0 ± 27.1 * #

KC	32.8 ± 3.1	43.6 ± 4.8 *	28.1 ± 2.5	36.6 ± 4.6

MCP-1	69.3 ± 5.3	85.3 ± 7.4	69.2 ± 7.4	85.6 ± 9.3

MIP-1α	18.5 ± 1.5	20.9 ± 2.7	20.6 ± 1.6	18.9 ± 1.8

IFNγ	1.8 ± 0.4	2.7 ± 0.6	1.9 ± 0.7	2.3 ± 0.5

IL-1β	6.6 ± 0.7	8.4 ± 1.1	7.6 ± 0.8	9.0 ± 1.1

IL-6	13.5 ± 4.9	8.9 ± 2.4	13.2 ± 9.0	11.3 ± 5.9

TNFα	3.0 ± 0.2	3.6 ± 0.4	3.1 ± 0.2	3.2 ± 0.3

IL-4	0.5 ± 0.3	1.1 ± 0.4	0.8 ± 0.4	2.0 ± 1.1

IL-10	7.0 ± 1.1	9.2 ± 1.0	7.1 ± 1.4	8.2 ± 1.3

IL-13	3.2 ± 1.7	11.7 ± 3.9 *	4.5 ± 1.7	6.9 ± 3.0

VEGF	7.8 ± 1.5	8.4 ± 2.1	10.9 ± 2.4	9.5 ± 2.8

Data presented as pg/mg protein, mean ± SEM. n = 8-12. * p < 0.05 for comparison against wt, # p < 0.05 for comparisons between hAPP vs. hAPP/*PARP-1^-/- ^*(ANOVA with Bonferroni correction). Differences were not statistically significant when corrected for comparisons between the 12 cytokines analyzed. RANTES and TGFβ were also measured, but values were below calibration limits.

### PARP-1 regulates Aβ-induced microglial activation in brain

**Figure 4:**
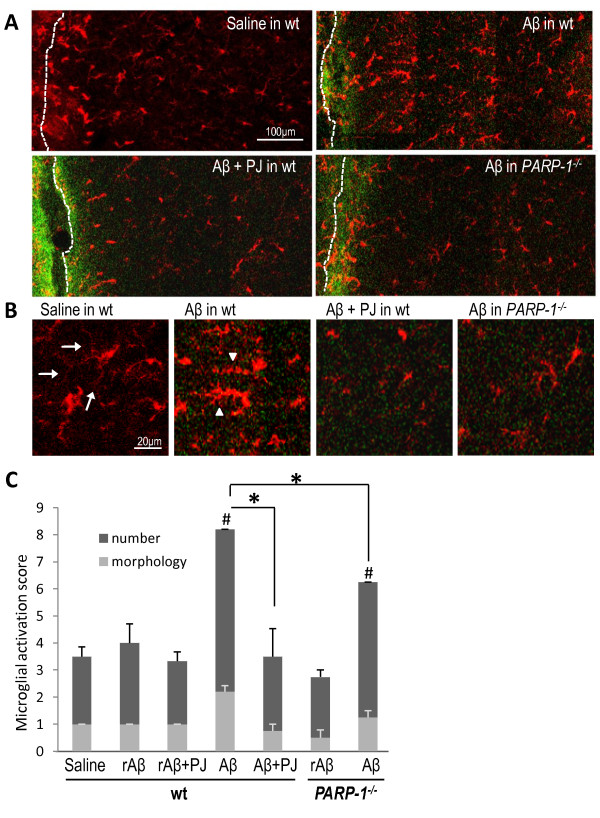
**PARP-1 regulates Aβ-induced microglial activation**. A, Photomicrographs show microglial morphology and Iba-1expression (red) in mouse hippocampus 6 h after stereotaxic injection of FAM-Aβ (green) or saline vehicle. The needle track is visible (and the edge is drawn) at the left-hand edge of each composite image. Microglial activation induced by Aβ injection was blocked by the PARP-1 inhibitor PJ34 (PJ) and in *PARP1^-/- ^*microglia. Injection of saline or reverse sequence Aβ (rAβ, not shown) produced microglial activation only at the needle track. B, High magnification views show ramified microglia with numerous long, thin, branched processes (arrows), and activated microglia with shorter, thickened processes (arrowheads). C, Quantification of microglia activation is presented in stacked columns presenting the scores for both microglial number and morphology. * p < 0.05; # p < 0.05 vs. saline; n = 4.

We considered the possibility that ageing hAPP_J20 _mice might express other factors, in addition to Aβ, that promote microglial activation. To directly determine the effects of PARP-1 deficiency on Aβ-induced microglial activation, we injected oligomeric Aβ directly into the hippocampus of wt and *PARP-1^-/- ^*mice. The Aβ injections induced soma enlargement and process retraction characteristic of activated microglia, and also increased microglial number in the area of injection (Figure 4). These changes were evident within 6 hours of the Aβ injections and were restricted to the area where Aβ was detected, i.e. ~500 μm from the injection needle track. In contrast, mice injected with vehicle (saline) or with a control, reverse-sequence Aβ (rAβ) showed microglial activation only in the immediate vicinity of the needle track lesion. Aβ injected into either *PARP-1^-/- ^*mice or wt mice treated with the PARP-1 inhibitor PJ34 produced substantially less microglial activation than Aβ injected into untreated wt mice (Figure 4).

### PARP-1 regulates Aβ-induced microglial activation in cell culture

**Figure 5:**
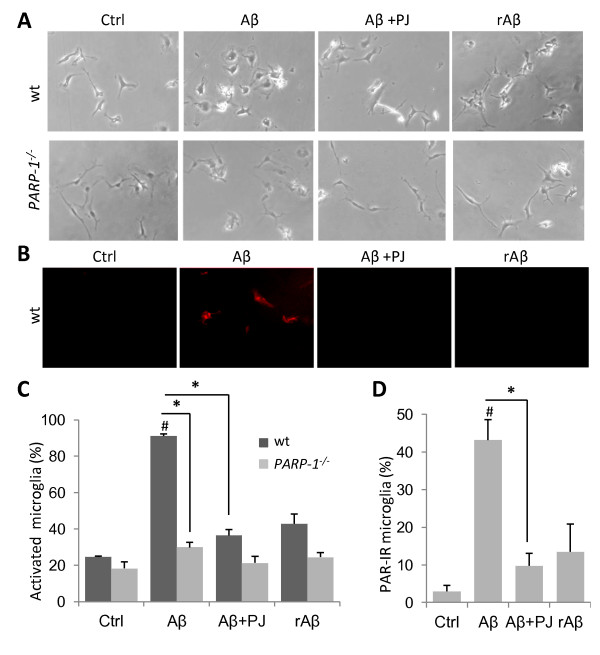
**Microglial activation by Aβ requires microglial PARP-1 activity**. A. Phase contrast images of cultured microglia. 24 hour incubation with 5 μM Aβ induces morphological transformation to the activated, amoeboid morphology in wild-type (wt) microglia. Microglial activation induced by Aβ was blocked by the PARP-1 inhibitor PJ34 (PJ, 400 nM) and in *PARP-1^-/- ^*microglia. Cells treated with the reverse sequence Aβ peptide (rAβ, 5 μM) remained in resting, ramified morphology. B, Immunostaining for poly(ADP-ribose), the enzymatic product of PARP-1, showed poly(ADP-ribose) production within 60 minutes of Aβ addition, blocked by PJ34. There was no poly(ADP-ribose) signal in *PARP-1^-/- ^*cells (not shown). C. Quantification of microglial activation. D, Quantification of poly(ADP-ribose) immunoreactive cells. * p < 0.05, # p < 0.05 vs. control. n = 3.

Results of the studies presented above suggest that the protective effects of PARP-1 deficiency are attributable to attenuated activation of *PARP-1^-/- ^*microglia. However, since *PARP-1^-/- ^*mice also lack PARP-1 in neurons, astrocytes, and other cell types, it is alternatively possible that the attenuated microglia response in these mice is secondary to effects of PARP-1 gene deletion in other cells. We therefore used cell cultures to assess the direct effects of PARP inhibition on microglia. Aβ stimulation of wt microglia induced transformation to either the fully activated amoeboid appearance or to a partially activated morphology, with enlarged soma and fewer, thickened processes. By contrast, *PARP-1^-/- ^*microglia retained the resting, ramified morphology, as did microglia of either genotype treated with vehicle or with the control peptide, rAβ (Figure 5). Microglial proliferation and viability were not affected by Aβ incubation (not shown). A rapid accumulation (within 1 hour) of poly(ADP-ribose) (PAR) was detected in Aβ-stimulated wt microglia, indicating enzymatic PARP-1 activity. The accumulation of PAR was blocked by co-incubation with the PARP inhibitor, PJ34 (Figure 5). PJ34 also blocked morphological transformation in microglia treated with Aβ exposure, supporting a requisite role for microglial PARP-1 activity in this process (Figure 5).

### PARP-1 regulates microglia - mediated Aβ neurotoxicity

**Figure 6:**
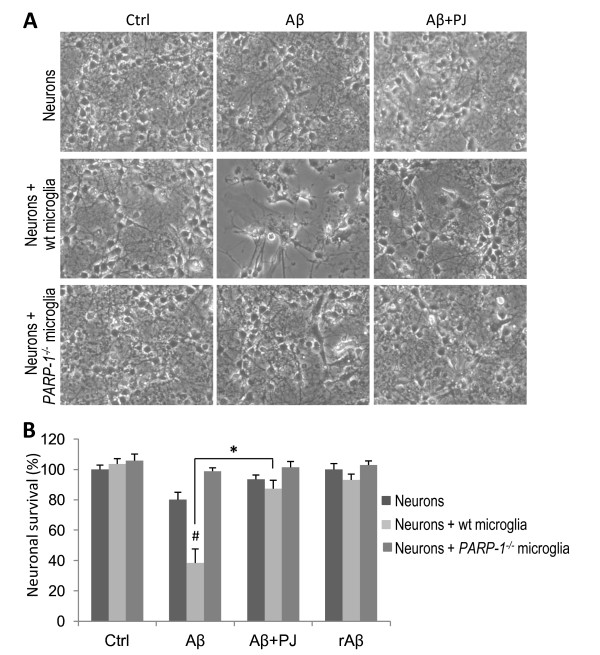
**PARP-1 regulates microglia-mediated amyloid β neurotoxicity**. A, Representative microphotographs from wt neurons cultured alone, with wt microglia, or with *PARP-1^-/- ^*microglia exposed to 5 μM of Aβ for 36 h. Aβ caused death of neurons cultured with wt microglia, but not in neurons cultured alone or with *PARP-1^-/- ^*microglia. rAβ produced no neuronal death (not shown). Aβ-induced death of neurons in co-culture with wt microglia was blocked by the PARP-1 inhibitor PJ34 (PJ, 400 nM). B, Quantitative assessment of neuron survival, expressed relative to neurons cultured without Aβ or microglia (control). *p < 0.005; # p < 0.05 vs. control. n = 3.

Microglial activation by Aβ and other stimuli can promote neuronal death [34,39-41]. We evaluated the role of PARP-1 in microglial neurotoxicity using neuron-microglia co-cultures. Twenty-four hours incubation with 5 μM Aβ caused no significant cell death in neuron monocultures, but killed more than 50% of neurons cultured with wt microglia. The microglia-mediated Aβ toxicity was abolished in cultures treated with the PARP-1 inhibitor, PJ34, and in wt neurons co-cultured with *PARP-1^-/- ^*microglia (Figure 6).

### PARP-1 regulates Aβ-induced microglial activation via NF-κB

**Figure 7:**
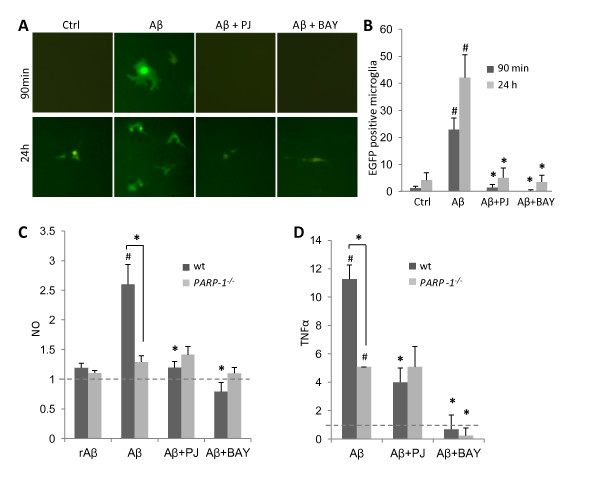
**PARP-1 regulates NF-κB - mediated gene transcription in Aβ-treated microglia**. A, Microglia were transfected with a reporter gene in which dEGFP expression is driven by the NF-κB p65 subunit. NF-κB transcriptional activity was evaluated after 90 min or 24 h of Aβ exposure. dEGFP expression induced by 5 μM Aβ was blocked by inhibitors of NF-κB activation (BAY 11-7082, 5 μM) or PARP-1 activation (PJ34, 400 nM). Quantified data are shown in panel (B). * p < 0.05 compared to Aβ, # p < 0.05 compared to control; n = 3. C-D, BAY and PJ34 also had parallel effects on microglial release of NO and TNFα, as assessed over 24 hours exposure to Aβ. Data are presented as a fold increase or decrease from control levels. Release from wt and *PARP-1^-/- ^*microglia was not significantly different under control conditions. * p < 0.005 vs. Aβ, # p < 0.05 vs. control. n = 3-4.

The transcription factor NF-κB is involved in many aspects of microglial inflammatory responses [42], and PARP-1 regulates the transcriptional activity of NF-κB [15,19]. Microglia cultures were transfected with an NF-κB-driven eGFP reporter gene [34] to evaluate the effects of Aβ and PARP-1 on NF-κB transcriptional activation in microglia. Aβ produced a large increase in the number of microglia expressing eGFP when assessed at either 90 minutes or 24 hours, and this increase was prevented by PARP inhibition (Figure 7A,B). Nitric oxide release and TNFα release are both regulated by NF-κB in myeloid cells [40,43]. Accordingly, microglial release of NO and TNFα were found to be stimulated by Aβ, and blocked by the NF-κB inhibitor, BAY 11-7082 [44]. The release was also blocked by the PARP-1 inhibitor PJ34 and in *PARP-1^-/- ^*cells (Figure 7C,D). PJ34 and BAY 11-7082 also reduced microglial release of NO and TNFα in the absence of Aβ stimulation although basal release was not reduced in *PARP-1^-/- ^*microglia (data not shown). Aβ stimulation also increased release of other NF-κB regulated cytokines (KC, RANTES, MCP-1and MIP-1β; Table 3). The magnitude of increase was reduced by PARP-1 abrogation, but the statistical significance was not reached or was lost after correction for the multiple group comparisons (Table 3).

**Table 3:** Cytokine levels in microglia cultures

	wt microglia			*PARP-1^-/- ^*microglia		
**Cytokine**	**Basal** **concentration**	**Fold change with treatment**		**Basal** **concentration**	**Fold change with treatment**	

		**Aβ**	**Aβ+PJ34**		**Aβ**	**Aβ+PJ34**

KC	0.78 ± 0.27	3.03 ± 1.5#	1.77 ± 0.67	0.39 ± 0.1*	1.17 ± 0.19†	1.08 ± 0.09

RANTES	1.46 ± 0.32	2.26 ± 0.86#	1.4 ± 0.27	0.76 ± 0.1*	1.69 ± 0.29#	2.36 ± 0.83

MCP-1	2.58 ± 1.07	1.81 ± 0.25#	1.15 ± 0.07†	1.06 ± 0.47	0.94 ± 0.18†	1.16 ± 0.33

MIP-1β	60.6 ± 41.9	1.91 ± 0.20#	1.44 ± 0.33	18.9 ± 4.6*	1.77 ± 0.28#	1.68 ± 0.19

IP-10	326 ± 113	0.36 ± 0.12	0.56 ± 0.10	43.4 ± 2.2*	0.48 ± 0.07	0.68 ± 0.21

IFNγ	5.63 ± 1.84	1.08 ± 0.13	0.74 ± 0.10	4.78 ± 2.26	3.14 ± 2.27	0.97 ± 0.68

IGF-1	11.1 ± 2.81	1.09 ± 0.02	1.93 ± 0.46	9.93 ± 0.23	1.17 ± 0.07	1.24 ± 0.17

Where indicated, cultures were treated with 5 μM Aβ, or 5 μM Aβ plus 400 nM PJ34 for 24 hours. Basal concentrations are pg/ml, normalized to microglial protein concentration; mean ± SEM. * p < 0.05 for comparison between wt and *PARP-1^-/-^*. Changes induced by the designated treatments are expressed relative to the basal concentration from each of 4 independent experiments (means ± SEM). # p < 0.05 for comparison between basal concentration and Aβ treatment. † p < 0.05 for comparisons against the Aβ treated wild-type cells (ANOVA with Dunnett's post-test). Differences were not statistically significant when corrected for comparisons between the 7 cytokines analyzed. IL-1α, IL-1β, IL-2, IL-3, IL-4, IL-5, IL-6, IL-9, IL-10, IL-12(p40 &p70), IL-13, IL-17 and GM-CSF were also measured but remained below calibration limits.

### PARP-1 modulates microglial trophic factor release

**Figure 8:**
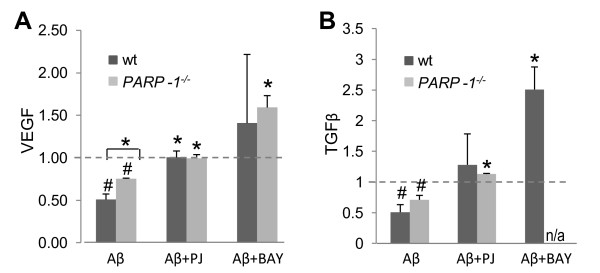
**PARP-1 modulates trophic factor release in Aβ-treated microglia**. Microglial release of VEGF and TGFβ were reduced during 24 hours of Aβ exposure. This reduction was attenuated in *PARP-1^-/- ^*microglia and in wt microglia treated with by inhibitors of PARP-1 (PJ34, 400 nM) or NF-κB (BAY 11-7082, 5 μM). Data are presented as a fold increase or decrease from control levels. Release from wt and *PARP-1^-/- ^*microglia was not significantly different under control conditions. * p < 0.005 vs. Aβ, # p < 0.05 vs. control. n = 3-4.

Activated microglia can also release, in addition to neurotoxic agents, several cytokines and trophic factors that can promote neuronal survival [8,45-47]. In particular, vascular endothelial growth factor (VEGF) and transforming growth factor β (TGFβ) are released by microglia [48-50] and have beneficial effects in experimental AD ([51,52], but see also [53]). Here, Aβ was found to reduce microglial release of both VEGF and TGFβ. This reduction was reversed by inhibitors of PARP-1 and NF-κB (Figure 8). These treatments also increased basal VEGF and TGFβ release (not shown).

### PARP-1 inhibition does not impair phagocytosis of Aβ peptides

**Figure 9:**
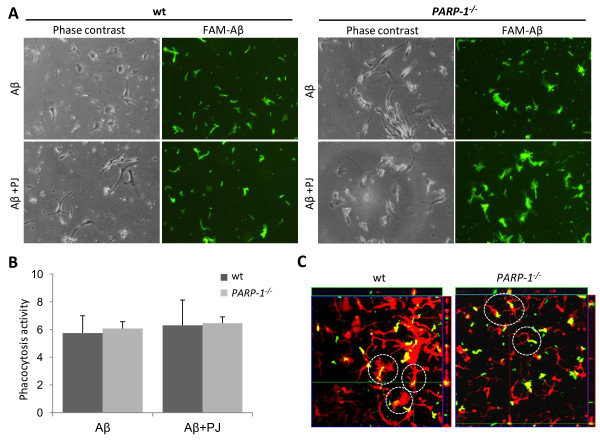
**PARP-1 abrogation does not impair microglial phagocytosis of Aβ**. A, Phase contrast and fluorescence images show the same field of microglia cultures. FAM-labeled Aβ (5 μM, green) is internalized by both wt and *PARP-1^-/- ^*microglia, with or without a PARP-1 inhibitor (PJ34, 400 nM). B, Quantification of microglial FAM-Aβ phagocytosis activity, assessed after a 24 h exposure (n = 3). C, Orthogonal confocal views of microglia (stained with Iba-1, red) and FAM-Aβ (green) after 24 h incubation. The *PARP-1^-/- ^*microglia do not assume a reactive morphology, but nevertheless engulfed the FAM-Aβ (yellow merge).

We examined the possibility that the reduced microglial activation produced by PARP-1 inhibition might also result in reduced clearance of Aβ peptides, using FAM-labeled Aβ. Cultured microglia rapidly engulfed and accumulated the FAM-Aβ peptides, and this was unaffected by PARP-1 inhibition or *PARP-1^-/- ^*genotype (Figure 9). Of note, *PARP-1^-/- ^*microglia with engulfed Aβ peptide maintained the resting, ramified morphology, unlike the wt microglia (Figure 9C).

## Discussion

Aβ, in addition to its direct effects on neuronal and synaptic function, may also stimulate microglial activation and pro-inflammatory responses in AD. Results presented here characterize the effects of PARP-1 on Aβ-induced microglial activation. hAPP_J20 _mice exhibited microglial activation, reduced hippocampal CA1 calbindin expression, and impaired novel object recognition at age 6 months, and all these features were attenuated in hAPP_J20 _mice lacking PARP-1 expression. Similarly, Aβ injected into mouse brain produced a robust microglial response, and this response was blocked in mice lacking PARP-1 expression or activity. Studies using microglial cultures showed that PARP-1 expression and activity were required for Aβ-induced NF-κB activation, morphological transformation, NO release, and TNFα release. PARP-1 expression and activity were also required for Aβ-induced microglial neurotoxicity. Conversely, PARP-1 inhibition increased microglia release of TGFβ and VEGF, and did not impair microglial phagocytosis of Aβ peptide.

Aβ injections into brain produced a robust microglial reaction localized to the area of Aβ diffusion. The local concentration of Aβ peptides produced by these injections is likely much higher than occurs in AD, and the sudden increase in Aβ is non-physiologic; however, the near-complete absence of Aβ-induced microglial activation in *PARP-1^-/- ^*mice or in wt mice treated with a PARP-1 inhibitor supports the idea that PARP-1 activity is essential for microglial activation in response to Aβ. Microglial activation in the hAPP_J20 _mouse was much less pronounced than that induced by Aβ injection, and interpretation of studies in the hAPP_J20_/*PARP-1^-/- ^*mice are complicated by the fact that neurons and other cell types in these mice also developmentally lack PARP-1 expression. Nevertheless, PARP-1 depletion reduced the number of activated microglia in hAPP_J20 _mice, in both amyloid plaques and non-plaque areas., while the total number of microglia was not affected. This finding together with the *in vitro *data demonstrates that PARP-1 abrogation does not affect viability or proliferation of Aβ-stimulated microglia. The comparable numbers of microglia in these analyses also suggests that PARP-1 depletion does not affect the migration of microglia or blood-born macrophages during Aβ stimulation.

Lesion and c-fos imaging studies suggest that the CA1 is involved in novel object recognition [54,55], whereas dentate gyrus lesions cause impaired spatial learning and memory [38]. Here, as previously reported [38], a loss of calbindin immunoreactivity was observed in the hippocampus of the hAPP_J20 _mice. Relative to the hAPP_J20 _mice, the hAPP_J20_/*PARP-1^-/-^* mice had less calbindin depletion in the hippocampal CA1, but not in the dentate gyrus. There is no obvious explanation for this regional difference, but this histological finding does comport with the mouse cognitive assessments, in which the hAPP_J20_/*PARP-1^-/- ^*mice performed better than hAPP_J20 _mice in the novel object recognition test, but not in the test of spatial memory.

NF-κB plays a major role in mediating Aβ-induced microglial neurotoxicity [34]. Results of the present cell culture studies indicate that effects of PARP-1 expression on microglial inflammatory responses are mediated, at least in part, through its interactions with NF-κB. PARP-1 abrogation prevented Aβ-induced NF-κB transcriptional activity, as evaluated with a κB driven eGFP reporter gene. In addition, pharmacological inhibition of NF-κB translocation reduced microglial NO and TNFα release to an extent comparable to that achieved with PARP-1 abrogation, and inhibitors of both NF-κB and PARP-1 have been shown to block microglial morphological activation [24,25]. A link between PARP-1 activation and NF-κB has been established [16,17,19,25]; however, PARP-1 also interacts with AP-1, NFAT, and Elk-1 [14,56,57], and PARP-1 interactions with these or other transcription factors may also regulate microglia responses to Aβ. Of note, PARP-2 and other PARP species also interact with transcription factors that regulate inflammation, and consequently the effects of PJ34 and other PARP-1 inhibitors could be mediated in part by these other PARP species [58].

Several secreted factors have been identified as mediators of microglial neurotoxicity, including TNFα and NO [40,59-61]. Results presented here show that Aβ-induced microglial neurotoxicity is PARP-1 dependent, an effect that may be attributable to the decreased release of both TNFα and NO observed with PARP-1 abrogation. In addition, Aβ-induced reduction of microglial TGFβ and VEGF release was attenuated by PARP-1 abrogation. Given that both of these factors suppress classical microglial activation [10], and TGFβ in addition promotes microglial phagocytosis and reduces Aβ accumulation in experimental AD [9], effects mediated by these trophic factors may be an additional mechanism by which PARP-1 influences brain response to Aβ.

Increased phagocytic activity is also a feature of microglial activation [4]. We therefore evaluated the possibility that PARP-1 inhibition could block microglial phagocytosis of Aβ, because this effect may be deleterious in AD brain. Results of these studies showed that PARP-1 activation does not block Aβ phagocytosis: levels of both total Aβ and Aβ_1-42 _were very similar in the hAPP_J20 _and hAPP_J20_/*PARP-1^-/- ^*mice, and uptake of Aβ by cultured microglia was unaffected by either PARP-1 deficiency or PARP-1 inhibition. These results are consistent with prior reports that minocycline, which is a potent PARP inhibitor [62], likewise does not block Aβ phagocytosis by microglia [63,64].

## Conclusions

The present study is, to our knowledge, the first to evaluate the therapeutic potential of PARP-1 inhibition in AD. The results show that PARP-1 inhibition attenuates Aβ-induced microglial activation and microglial neurotoxicity. PARP-1 inhibitors are entering clinical use for other conditions, and compounds such as minocycline with potent PARP-1 inhibitory effects are being explored in AD models [65-67]. Results presented here support the rationale for this approach to suppressing neurotoxic aspects of Aβ-induced microglial activation in AD.

## Competing interests

The authors declare that they have no competing interests.

## Authors' contributions

TMK designed the experiments, performed most of the experiments and collected the data and prepared the manuscript, SWS performed hippocampal Aβ injections, YH participated in the microglia culture experiments, AEB participated in hAPP_J20_/*PARP-1^-/- ^*mice generation, CE participated in the cytokine assays, SJW perfused and collected the brain tissue from the in vivo experiments, CW performed Aβ ELISAs, SHC participated in the hAPP_J20 _mice immunostaining process, LG participated in design of hAPP_J20_/*PARP-1^-/- ^*mice experiments and manuscript preparation, RAS participated in experimental design and preparation of manuscript. All authors read and approved the final manuscript.
